# "The Hospital" Nursing Mirror

**Published:** 1897-12-11

**Authors:** 


### "The Hospital" Nursing Mirror.

Being the Nursing Section of "The Hospital."

[Contributions for this Section of "The Hospital" should be addressed to the Editor, The Hospital, 28 & 29, Southampton Street, Strand London, W.C., and should have the word "Nursing" plainly written in left-hand top corner of the envelope.]

### News from the Nursing World.

### THE ROYAL NATIONAL PENSION FUND FOR NURSES.

Mr. Everard Alexander Hambro has consented to become Chairman of the Royal National Pension Fund for Nurses, vacant through the lamented death of the late Mr. Walter Burns. Mr. Hambro is one of the four merchant princes who gave £5,000 each to enable the Pension Fund to be established. There is no man who is more deservedly or more highly respected in the City of London than Mr. Hambro, and his acceptance of the chairmanship is as important to the Fund as it is honourable to himself, having regard to the many important claims upon his time and energy. Mr. Hambro was unanimously elected chairman at the last meeting of the Council of the Fund. The Secretary of the Fund asks us to remind nurse members that their subscription of 1s. for the ensuing year to their benevolent fund is now due. We feel sure subscribers will not need written reminders, as that will reduce their contribution to 11d.; and to prevent this, and that the benevolent fund may get every penny possible, we are printing this notice in our columns, and hope that those nurses who read the "Mirror" will kindly tell those who do not. Last year the nurses' shillings brought in £70. We hope this year the number of subscribers will be still greater.

### THE WORKHOUSE INFIRMARY NURSING ASSOCIATION.

The meeting of the Workhouse Infirmary Nursing Association on December 3rd, at St. Martin's Town Hall, practically closed the association's public career in its present form. We cannot let the occasion pass without expressing our grateful recognition of the valuable work done by the association, not only in training and supplying nurses to the workhouses, but in educating the opinion of the public and boards of guardians in their duty towards the infirm paupers. It is out of the question to suggest any possible course of action to an association which has shown such a thorough knowledge of the vital problems with which it deals, and of the tact necessary to success in handling them; but we must express our regret that the public boards of guardians and nurses are deprived of the services of this most experienced body at a very critical moment. We cannot, however, fail to gather hope from the facts that the executive committee still exists, and expresses itself ready for work when the opportunity arrives, that the near future will see some new but equally useful development. If the spirit exists, which it undoubtedly does, it will certainly express itself sooner or later in a practical form.

### SALE OF WORK AT THE ORTHOPÆDIC HOSPITAL.

The Duchess of Marlborough opened the annual sale of work at the Orthopædic Hospital on the 3rd inst. in aid of the Extra Comfort Fund. The stalls were attractively arranged in the out-patients' department, chiefly with useful and ornamental garments. Specimens of really beautiful needlework were to be found on more than one, whilst some doyleys of drawn silk and lace stitches were worthy of a fine-art needlework exhibition. The Duchess and Lady Farquhar, both of whom take a great interest in the hospital and often visit it, bought largely, and many others present did the same, so that there is a bright outlook in the way of extra comforts for the forthcoming year. The balcony, on to which the children are carried on summer days, is the gift of this fund last year. Clocks, too, have been placed by it in some of the wards, and deserving patients are helped in many ways. The hospital is a mixture of old and new, and though there is an atmosphere of good management and comfort throughout, and excellent work is done in the quaint old wards, yet picturesque appearances do not always make up for modern conveniences.

### QUEEN CHARLOTTE'S NURSES.

The nurses at Queen Charlotte's are divided into two distinct classes—those who are on the permanent staff, and those known as pupil nurses, who come and go every three or six months. Those on the permanent staff are housed in the hospital, and a new storey is being added at the present time in order to accommodate them. The pupil nurses, on the other hand, have hitherto lived at some little distance away. The authorities are now issuing an appeal for money to build a new home for them, as the leases of the houses occupied have run out, and some difficulty has been experienced even in procuring a site whereon to build. As there is not a suitable house in the neighbourhood available for the purpose, this is the only alternative. The hospital also suffers much from the lack of a good laundry. The uniform of the nurses is white piqué, a charming dress when fresh, but with bad and destructive work in the washtub it looks tumbled and soiled in a very short space of time. The infants' garments are not well washed and ironed, much to the annoyance of the nurses, who like to see the babies and patients in snowy linen, whilst the destruction to flannel is serious. It is next to impossible to get washing done satisfactorily in town, and the authorities would find it a very practical economy if they started a laundry of their own.

### THE DALGLEISH NURSES' HOME.

Sir William Dalgleish, in the presence of a large gathering of friends, opened the new nurses' home of the Dundee Royal Infirmary, of which he is the donor, on November 30th. This is the second nurses' home belonging to the infirmary; the former, which is now inadequate to accommodate the increased staff of the infirmary, was the gift of Mrs. Gilroy some years ago. The new building has a separate room for 37 nurses separate sitting-rooms for the assistant matron, the sisters, and the nurses, and a library. It is furnished comfortably, and the blinds and wall-papers are cheerful. It is connected with the main building by a covered corridor. Sir William Dalgleish, in opening the home, dwelt upon the importance of a comfortable and cheerful dwelling to all workers, and when he found out two or three years ago that two and sometimes three of the infirmary nurses occupied the same room, he determined to put the matter right. Great care has been bestowed upon the building and equipment, in order that it shall fulfil all modern requirements.

### SLAVES IN THE WORKHOUSE.

Miss Brigs, a nurse in the Cockermouth Workhouse, resigned her post, stating plainly her reasons for doing so. "I did not," she wrote, "when I accepted the appointment, expect to do duty night and day, and I do not consider the food good enough to wish to remain, as, after being up the greater part of the night, I had to pay 2d. for an egg for my breakfast, as there was not sufficient bacon provided to last the week." Upon inquiry, it was found that the nurse had lately been up two whole nights in one week. The one good point about this case is Miss Brigs' courage in stating her reasons for resignation. If nurses would always do so, many of the wrongs they suffer from would be righted. Guardians, even supposing they fail to see why modern nursing in the workhouses under their control should be brought so nearly to the pitch of perfection, cannot be so inhuman as to work a woman night and day and then to make her pay for the deficiency in their food supply when the fact is plainly stated; nor, if they would, is it to be for one moment supposed that public opinion will permit them to do so.

### THE OPENING OF BROUGH NURSING HOME, PAISLEY.

Part of the munificent bequest of the late Mr. Peter Brough, of Paisley, has been expended in building a nurses' home in that town. It was opened by the Princess Louise Marchioness of Lorne on November 30th. The opening ceremony was very short, the speeches usual on such occasions being made later at the luncheon. Lord Lorne returned thanks for the Marchioness and himself; and, whilst complimenting the inhabitants on their handsome halls, he suggested that the art of painting should not be neglected in their adornment. The district nurses have been working in Paisley for the past thirteen years. About four years ago their association was affiliated with Queen Victoria's Jubilee Institute. The staff now in possession of the new home consists of a superintendent and six nurses. Mr. Peter Brough's estate also provides a maintenance fund.

### NEW NURSES' HOME, GLASGOW.

A much-needed nurses' home for the accommodation of the staff employed at the Sick Children's Hospital at West Graham Street, Glasgow, was opened on November 25th by Lady Blythswood. Hitherto some of the nurses have lived in a small cottage situated in a lane at the back, and six of them at the hospital. The new home provides bedrooms, dining and sitting rooms, kitchen, &c., for the nurses, and a board-room for the Ladies' Auxiliary Committee. The quarters occupied by the nurses up to the present in the dispensary will be used for specialist work (dental, aural, ophthalmic, &c.), lectures, and demonstrations. The extra space in the hospital permits a room to be reserved for sickness amongst the nurses. Several gentlemen congratulated the hospital on its new acquisition, and Lady Blythswood made a few sympathetic remarks in declaring the home open.

### CHESTERFIELD'S PROSPECTIVE NURSES.

Chesterfield desires to raise £350 in order to found a nurses' home. Of this sum £150 is promised, and £100 offered upon the completion of the total. The Chesterfield Amateur Dramatic Society have given a successful entertainment in aid of the fund, and probably the rest of the money will soon be forthcoming. The scheme of the new Victoria Home is fully drawn up, and is very much the same as that adopted by the majority of such institutions, but there is one clause that reveals a wrong principle. Clause No. III. runs—"To maintain out of the profits one or more district nurses for the free service of the poor in their own houses." £350 is a very small sum indeed; it represents a rental of about £15. In this home a staff of fully-trained nurses is to be run for the benefit of the district, which is to pay the home full fees for their services. The profits are to be devoted to keeping one or two district nurses, and the yearly cost of these is £80 a year. The whole thing is absurd. Why should nurses, for the sake of residence in a paltry £15 a-year cottage, give the district their earnings to the tune of from £80 to £120 tper annum? It cannot be done.

### NURSES' HOME AT KINGSTON.

A Nurses' Home forms part of the new buildings which are being erected at Kingston to serve as the Union Infirmary. The home stands apart from the infirmary, and is to contain 31 bedrooms, together with reading-room, sitting-room, dining-room, a room for the sisters, and bath-rooms, and the usual offices. At the ceremony of laying the foundation-stone last week, a great deal of interest was created by the presence of "Granny" Stevens, the centenarian of the workhouse, who much enjoyed the festive occasion, which she viewed from the platform.

### NORTH SUSSEX NURSING ASSOCIATION.

The scheme set afoot to commemorate the year by the Earl and Countess De la Warr is the formation of a nursing association for the villages of northern Sussex. Two grades of nurses will be employed, the highly trained district, and the cottage nurse.

### GLASTONBURY NURSING HOME.

Mrs. J. A. Porch laid the foundation-stone of the "Victoria Nursing Home," the town's commemoration of this year's rejoicing. Mr. Stanley Austin practically gave the site, and £300 of the necessary £400 is already subscribed.

### SHORT ITEMS.

There is happily no further need of the Nurses' Stores at Maidstone. The few patients still remaining will have their wants supplied from the Mayor's Stores at the Corn Exchange.—At the final examination for nurses at the Royal United Hospital, Bath, Nurse Pidgeon obtained 351 marks, and is gold medallist; Nurse Marian Jones, 338 marks, and is silver medallist; Nurse Webber, 337 marks: Nurse Edith Joseph, 325 marks; Nurse Edmunds, 324 marks; and Nurse Rhoda, 316 marks. The full number of marks was 400.

### Lectures to Surgical Nurses.

By H. A. LATIMER, M.D. (Dunelm), M.R.C.S. of Eng., L.S.A. of London, Consulting Surgeon, Swansea Hospital; President of the Swansea Medical Society; Lecturer and Examiner of the St. John Ambulance Association, &c.

### XX.—THE "SETTING" OF FRACTURES: THE COMPLICATIONS ATTENDING THEM.

HAVING instructed you as to the various kinds of fractures to be met with, I will pass on to consider the mode adopted in "setting" them, the results of failure in union, the complications attending them, and the processes by which they are repaired. In the majority of cases coming before your notice these injuries will have been attended to before you see them, and in all cases the careful adjustment of the broken bones will be undertaken by the surgeons; but it may be that you will have to render "first aid" to the injured before the arrival of the surgeon, as it is a most important matter that precautions should be adopted directly a bone has been broken, to prevent a simple fracture being converted into a complicated or compound one. The "first aid" consists in adopting an appropriate treatment for a person who has been stunned or bleeding, and in applying some form of splint to restrain the action of muscles, and to keep the bones steadily in their place. The teaching you shall receive when I deal with the unconscious state, and the instruction which you have already received about hæmorrhage, will enable you to attend to these points, so I will pass on at once to the last one alluded to above, of attention to the fracture itself. I have told you that it is of the greatest importance that the bone which has been broken shall be at once encircled or supported by some kind of splint or restraining apparatus, so that a simple fracture may not be converted into a compound one. It matters little what this apparatus shall consist of, provided it is one which will serve its purpose of keeping the bones together. Pieces of wood, sticks, umbrellas, backs of books, stout newspapers, or similar handy things, serve well as improvised splints; and after some two or more of these have been placed over the injured part, and have been secured there by handkerchiefs or bandages, the patients can be removed to their houses, or to a hospital, for further and more scientific treatment. Where it is suspected, but not proved, that a bone has been broken, it is the wisest thing to proceed as if that injury has taken place, for you cannot do any harm by taking the graver view, whereas great mischief may result from your neglecting to adopt supporting measures. There is no doubt but that the sooner a fracture is "set" the better it is for the patient, but it is not always possible to apply the full treatment at once, so much swelling occurring about the part that it is difficult to make out exactly what has happened, or to put on splints that shall serve as more than temporary supports. But it is just these temporary splints which are called for at your hands, or at the hands of unskilled witnesses of the accident a person has sustained.

Proceed in this way when rendering "first aid." If an arm or a leg has been injured and blood is flowing from any wound there, rip up the garments covering it, by running a knife along their seams, and see from whence the blood flows. Then proceed without delay to stop this bleeding. Keep the patient lying down, and now gently insinuate the palms of your hands under his injured limb and hold it at points a little above and below the seat of a suspected fracture; now make gentle movements with the hands, and see if you can detect grating or a movement in parts which ought to be immovable. If you determine to apply splints, select what you want, and have them ready for use; now get someone to hold the damaged limb very steadily at some distance from the injured part, and, seizing the other end of the limb with one of your hands, gently pull upon it to correct any deformity; and, whilst maintaining a hold on the limb with one hand, after having satisfied yourself that it is in a straight line, take up the splint with the other hand and apply it to one side of the limb, and holding it there with this hand, have its fellow placed opposite to it by your helper. The latter may now leave his place and hold the splints in position for you whilst you apply bandages to them. Your patient is now in a condition to be taken away from his place of injury. In the case of an arm, after placing a sling round the neck, and under the forearm, he can walk away; if his leg be broken he must be carried on some sort of stretcher. If the thigh bone should have been broken you will have to place one long splint on its outer side reaching from the armpit to below the foot, and another shorter splint opposite to the first, extending from the juncture of the leg with the body; after having bandaged these you had better bind the two legs together to make sure of his being kept quiet and to afford additional support to the injured limb. In the case of a protruding bone, in a compound fracture, you should attempt to withdraw it from the wound by steadily pulling upon the limb, as I have described before. This is called "making extension," if you succeed in effecting a replacement. Then dress the wound, apply splints, and bandage.

The collar bone is very frequently broken by direct and indirect violence; the fracture is not a difficult one to detect because the bone is to be felt and seen along the whole of its course. The resulting deformity is great as the bone is pulled upon by the weight of the arm, which is no longer kept in its proper place owing to the yielding of the outer fragment of the broken bone. To make sure of what has happened, expose the chest to view, and, standing opposite the patient, run your fingers along both collar bones so as to compare them. If one of them is broken you will note that it is different in shape to its fellow, and the sufferer will be seen to be supporting the arm at the injured side with the hand of the sound side; or if he is not doing this, his shoulder will have fallen forwards and downwards. Render "first aid" by lifting the fallen shoulder into place, by pushing up and supporting the elbow to that side; then place a pad made of a good sized handkerchief into the arm-pit, bind the fore-arm and support it with an arm sling tied at the nape of the neck; and, so that everything may be kept quietly in place, apply a bandage round the arm, to keep it to the side, or fix it in the same way with a handkerchief.

Broken ribs are revealed by the pain which takes place as breathing is carried on. They are often difficult to detect for a certainty, but this is a matter of little importance to you, for wherever a stitch accompanies the act of breathing, it is an advisable thing to apply a tight bandage round the chest so as to restrain the movements of the ribs.

The remaining fractures which I shall speak of are ones in which you can do little in rendering "first aid," as the injuries caused by them are so severe that they have to be attended to by surgeons. When the skull has been broken the fracture will have more or less grave consequences, according to the part which has been injured. Should it have been broken on the vault, bleeding may be met with, but here, as in the case of fractures within the skull at its base, the grave symptoms which ensue are due to injury to the brain. These symptoms consist in a greater or lesser degree of stunning and unconsciousness, followed in the case of the graver injuries by paralysis or by speedy death. When the vault of the skull has been broken there is especial liability for the fractured bones to be driven in to the brain, which lies underneath them, and then we have to deal with what is called "compression of the brain." The symptoms of compression of the brain are unconsciousness, altered conditions of the pupils of the eyes—one pupil being different in size to its fellow—deep sleep with snoring breathing, perspiration, and a slow, but full, bounding pulse. To relieve such a state of affairs the surgeon performs the operation of trephining—an operation by which, after the broken bone has been exposed by turning the scalp aside, a portion of it is removed with a circular saw, and so the pressure which is being exercised upon the brain is removed. Fracture of the base of the skull also leads to a condition of unconsciousness; the special symptoms which mark it are, in addition to the ones I have described to you, the draining away of watery fluid from the ears and nose. So far as your duties are concerned in nursing these people, they consist in attention to the general details of nursing, to the exercise of scrupulous care in attention to the skin, to prevent the formation of bed-sores, and to the carrying out of the special directions with regard to diet and quiet which will be enjoined by the surgeons. Any of the bones of the face may be broken by direct violence, the lower jaw frequently suffering in this way. You will be expected to prepare suitable bandages for putting up such a case; these bandages are constructed with flannel or lint, and are made by tearing a bandage to within four inches of its centre, so that it forms four tails. The centre of this bandage is placed over the point of the lower jaw, two ends are carried up along the sides of the jaw and tied above the head, whilst the other two tails are brought to the back part of the head, are there crossed, brought back over the forehead, and tied. These fractures of the jaw are so difficult to adjust and keep in place that it is often necessary to fix them by moulded splints of gutta-percha, or even to call in the assistance of the dentist to contrive some appliance for effectually securing them.

### Royal British Nurses' Association.

SIR JOHN LUBBOCK'S LECTURE.

ON December 1st Sir John Lubbock lectured before the Royal British Nurses' Association, taking "Ants" for his subject. He began by describing his method of observation. First, he made the ants so comfortable that they lived as nearly as possible under natural conditions. The life of ants was much longer than had been supposed. He had kept many for several years, and two queens in his possession reached the age of fifteen years; indeed, they were by far the oldest insects on record. Ants watched over their young with a skill and tenderness which, he said, not even the Royal British Nurses could excel. They never quarrelled. No one had ever seen a dispute between two ants belonging to the same nest, yet they were very brave and defended their homes like little six-legged Leonidases. Unlike the so-called higher animals, they never turned on a weak or wounded companion. One of his ants came into the world a cripple, but she was carefully tended and fed by her companions for months. All the ants of a community know one another, but they will not tolerate a stranger, even of the same species, in the nest. Sir John separated a nest into two halves, and found that even after eighteen months those separated recognised their old companions. It had been suggested that ants recognised one another by a sort of pass word, but this was not the case. Sir John made fifty ants quite drunk and incapable. He then put them near a nest to which twenty-five of them belonged. These twenty-five were carried back into the nest, where, no doubt, they slept off the effects of their involuntary debauch; the other 25 were thrown into the moat which surrounded the ants' park. The senses of ants differ in many respects from ours. They probably hear sounds which are inaudible to us, and see the ultra-violet rays which are invisible to us. Some species keep slaves, and some have even lost the instinct of feeding, and will starve by themselves even if provided with food. Sir John, however, found that they would live for weeks if they had a slave for an hour a day to feed and clean them. Several species keep aphides, which they milk like cows, and Sir John found that in the autumn they collect the eggs of the aphides and keep them all through winter, though they are of no use, and the young aphides hatched from them give none of the sugary fluid till the following May or June, so that the ants show more thrift and forethought than many human beings. Their instincts, though so wonderful, were very limited; but yet, when we watched ants building their nest, feeding their young, defending their homes, making roads, tending their domestic animals, and in some cases their slaves, it was difficult to suppose that they were mere unconscious automatons; and though their mental powers are no doubt greatly inferior to ours, the difference is probably not so much in kind as in degree.

### Appointments.

### MATRONS.

MEMORIAL COTTAGE HOSPITAL, ST. ANDREWS, FIFE.—Miss Jessie Torrance, who was trained in Paisley Infirmary and Fever Hospital, and afterwards nurse-matron at the Paisley Small-pox Hospital, and superintendent of the Greenock Hospital fever wards, was appointed Matron of the St. Andrew's Memorial Cottage Hospital on December 7th.

NORTH LONSDALE HOSPITAL, BARROW-IN-FURNESS.—Miss Ruth Summers, who has been ward and theatre sister, and latterly night superintendent at Macclesfield Infirmary, has been appointed Matron of the above institution. She was trained at Manchester Royal Infirmary.

BRIDGWATER INFIRMARY.—Miss Mary E. Bellamy, who was trained at East Dulwich Infirmary, and afterwards nurse at the Children's Infirmary, Liverpool, and the Leeds Hospital for Women and Children, has been promoted from being sister at Bridgwater Infirmary to be Matron.

### Where to Go.

ROYAL BRITISH NURSES' ASSOCIATION.—We are requested to announce that the annual conversazione of the Royal British Nurses' Association will be held at the Institute of Painters in Water Colours, Piccadilly, on Wednesday, December 15th, at half-past eight p.m. An attractive programme of entertainments, including a performance of scenes from "The Geisha" and a concert, are in course of preparation. Mr. David Devant has kindly consented to give a selection of his magical problems, and arrangements have been made with the Electrophone Company whereby the visitors will be placed in direct electrical communication with several of the leading London theatres and places of entertainment. The various railway companies have kindly promised to supply third class return tickets to London and back extending from December 14th to December 20th at a single fare for the double journey on presentation by members of tickets for the conversazione. Tickets, both for guests and members, are to be obtained from the Secretary, 17, Old Cavendish Street, W.

COLONIAL NURSING ASSOCIATION, IMPERIAL INSTITUTE, S.W.—The first annual general meeting of this association will be held at the Imperial Institute on Monday, December 13th, at three p.m., when the Right Hon. Lord Loch, G.C.B., G.C.M.G., will preside. An account will be given of the work of the association during the past year, and those interested are invited to attend.

### The Dangers of Nursing Syphilitic Cases, and How to Avoid Them.

SYPHILIS is a disease which, in the ordinary course of events, nurses do not see very much of and are not taught much about, the disease being still more or less obscured by the false modesty so prevalent in this country. Yet it must be obvious that a nurse should be acquainted with the main facts of the disease, especially as regards the questions of infection, both for her own sake and for that of her patients; for she may at any time be called upon to attend a patient suffering from this disease, and if not careful may contract it herself. For the disease may be, and too often is, contracted by doctors and nurses in the execution of their duty.

Syphilis is a contagious disease which may be acquired or hereditary.

A.—ACQUIRED SYPHILIS.

This is divided into three distinct stages, primary, secondary, and tertiary.

*Primary Syphilis.*—This consists in the formation of a sore at the point of inoculation, viz., the point where the poison enters the body. This is commonly on the external organs of generation. In cases of accidental inoculation it usually occurs on the fingers, as in the case of doctors or nurses, who become infected through handling syphilitic sores. In other cases it occurs on the lips, having been caught by kissing a person affected with syphilitic disease of the mouth or tongue. This primary sore soon becomes hard, and the nearest lymphatic glands become almost simultaneously hard and enlarged. In women the primary sores are more often soft than hard. The usual time of appearance of the primary sore is from four to six weeks after the time of inoculation.

*Secondary Syphilis.*—Secondary symptoms occur usually within two or three months. The first signs are rashes, inflammation of the tonsils, fauces, and soft palate, and enlargement of the lymphatic glands at the back of the neck. Where the rash is situated in parts naturally moist, the spots develop into moist, flat papules called condylomata, which are very characteristic of the disease. The throat and mouth often become affected with similar moist greyish white patches called mucus tubercles, which often become ulcerated.

*Tertiary Syphilis.*—This may occur within a year of the primary sore, but is usually delayed several years, and in cases which have gone through a thorough course of treatment often does not occur at all, or at any rate is very slight.

B.—INHERITED SYPHILIS.

This is syphilis inherited by the child from one or both parents affected with acquired syphilis. The majority of children infected with syphilis in this way are either prematurely born dead, or stillbirths. If the child is born alive it is usually of healthy appearance, but in a few weeks begins to waste and develop the characteristic signs of the disease. These are (1) "snuffles," due to inflammation of the mucous membrane lining the nose. This may interfere with the growth of the nasal bones, and give rise to the flat-nosed condition we so often see in adults who have suffered from congenital syphilis in their infancy. (2) The formation of a copper-coloured rash on the buttocks and between the thighs. (3) Condylomata about the anus and between the buttocks. (4) The wasted "old man" appearance, with sallow coloured skin and feeble cry. (5) Extreme wakefulness at night. These are the chief signs in the infant. Sometimes there are mucous tubercles and ulcers in the mouth, but these are not so common as in acquired syphilis.

In older children, from five to twelve years or even older, other characteristic symptoms may appear. The chief of these are (1) a notched and pegged condition of the permanent teeth (the milk teeth are not affected); (2) inflammation of one or both corneæ, causing a blurred and discoloured condition of the eye, with pain on facing the light. This affection of the eyes is known as interstitial keratitis, and is characteristic of inherited syphilis; (3) periostitis of the long bones; (4) synovitis of the knees; (5) mucous patches in the throat. These are the chief symptoms of the later stages of inherited syphilis. Other affections may occur, but they are not so common.

THE QUESTIONS OF CONTAGION AND INFECTION.

1. *Acquired Syphilis.*—Syphilis can only be contracted by direct contact, *i.e.*, it is contagious, and not infectious like scarlet fever or measles, which are conveyed through the air. The contagious sores of acquired syphilis are only the primary and secondary ones; tertiary sores are not contagious. The most contagious sores are the primary, and the condylomata and mucous tubercles of the secondary stage. The discharge from these sores is very contagious, and if it gains access to the system through a crack or abrasion in the skin will develop there a primary syphilitic sore, which will be followed in due course by secondary syphilis. All other secondary sores may be contagious, but in a less degree than those mentioned above.

2. *Inherited Syphilis.*—The dangers of contagion from inherited syphilis have been exaggerated, and it is comparatively uncommon for contagion to occur from such cases. However, it is well to bear in mind that condylomata and ulcers of the mouth in syphilitic infants are contagious. This is especially to be remembered when we consider that some infants and children contract acquired syphilis, and the nurse may not be in a position to know whether she has to deal with a case of inherited disease or of syphilis acquired by an infant accidentally after birth. Because an infant has the disease it is not safe for the nurse to assume that it has been inherited (apart from other information given her by the doctor); it may have been acquired, and the two are very different as regards contagiousness.

HOW TO AVOID CONTAGION.

This to a nurse brought up in modern antiseptic ways is very simple. If she has charge of a case of syphilis under a doctor, she must ascertain from him which sores are contagious. If she has any cracks or abrasions in the skin of her fingers they must be covered up with wool and collodion and a finger-stall before touching any primary or secondary sore, and the finger-stall must be disinfected in 1 in 20 carbolic lotion before being used again. If the fingers are free from cracks it is sufficient to rinse them well in 1 in 1,000 perchloride of mercury, or 1 in 20 carbolic lotion. All drinking and eating utensils used by a patient suffering from syphilis in a contagious state must be disinfected well. The same, of course, applies to all instruments, tongue depressors, &c. Boiling is the best plan for all these things, and is easily done in a large saucepan.

In conclusion, a few words may be said with regard to the suckling of infants with inherited syphilis. In all cases where possible the mother of a syphilitic child should suckle her child unless she has cracked nipples or the infant ulcers or sores about the mouth or tongue. The reason of this is that a mother who gives birth to a syphilitic child by a syphilitic father as a rule remains free from the disease (provided, of course, she did not have it before); in fact, she becomes inoculated against the disease through her child in a way analogous to vaccination against small-pox. But such a state of affairs is not constant, and occasionally a mother becomes infected with active syphilis through suckling her child, and acquires a primary sore on the nipple, infected from the sores in the mouth of the child. Therefore it is the rule not to allow the mother to suckle the child if she has cracked nipples, or if the child has sores in the mouth. It is out of the question for permission to be given to a healthy wet nurse to suckle a syphilitic infant, for although contagion from such cases is uncommon, yet it sometimes occurs, and if it does so the result is disastrous.

### Invalid Cookery.

### RECIPES FOR FISH DISHES.

WHITING IN OYSTER MILK.—This fish being so easily digested, it is invariably recommended as the first fish for the convalescent. Plainly boiled or steamed renders it rather insipid, but cooked by the following recipe it will prove most palatable and appetising. Procure a perfectly fresh whiting, wipe it with a clean cloth, then put it in a stewpan with half a-pint of warm milk, a pinch of salt, four peppercorns, and a tiny piece of mace. Cover the fish with a piece of paper, and put the lid on the stewpan, bring the milk to the boil, then simmer the fish slowly for 15 minutes or more, according to size; about 10 minutes before serving, throw in six bearded oysters; when the fish is cooked dish up on a hot dish, brush it over lightly with a little warmed fresh butter. Strain the milk and pour it round. Sprinkle the fish with finely chopped parsley. The oysters are likely to be hard, so they should not be served.

FRIED WHITING.—French frying is the best for invalid cookery; it is a very simple method, only requiring a deep iron stewpan about half full of clarified beef or mutton fat. Pork lard must never be used, as anything fried in it is most indigestible. The fat must reach boiling point, this is known by a faint blue smoke rising off the surface, and the fat being perfectly still, then plunge in whatever is to be fried. In this recipe it is a fresh whiting. It must first be wiped with a clean cloth, then rubbed with flour, brushed over with whole beaten up egg, and dipped in freshly-made bread crumbs that have been rubbed through a wire sieve. Place the fish in a frying basket and cook a nice brown colour, take up on kitchen paper. The crispness of the crumbs will entirely depend upon the fat being hot enough. Place the whiting on a dish paper on a hot dish and garnish with sprigs of parsley.

WHITING SOUFFLÉ.—Scrape the meat off a medium-sized whiting, chop it well, and rub it through a wire sieve. Put the purée in a basin and to two ounces add the yolks of two eggs, a little salt and coralline pepper, a quarter of a pint of whipped cream; stir in very gently the whites of two fresh eggs that have been whipped with a pinch of salt till they are quite stiff. Butter a soufflé dish and surround it with a band of buttered paper two inches above the edge, stand it on a tin, pour in the mixture, and bake the soufflé in a moderate oven for twenty minutes. When cooked remove the paper and fold a d'oyley round the dish; serve as quickly as possible.

FILLETS OF PLAICE.—Nicely cooked this fish is excellent, and considered by some to be lighter food than sole. Remove the fillets from a fresh plaice by keeping the knife close to the bone and working the fillet off. Turn the skin side down on the board, and remove it by keeping the knife close to it; the flesh will leave it quite easily. Bat the fillets out with a knife dipped in cold water, trim them neatly, and put them into a buttered tin, sprinkle a little lemon juice over each fillet, and a little salt and pepper; put a tablespoonful of water into the tin, and cover the fish with a buttered paper. Cook in a moderate oven for ten minutes, dish up neatly, and keep hot while the sauce is made. Put the liquor from the fish in a small saucepan, make it hot, and drop in by tiny pieces half an ounce of butter, a few drops of carmine, and a little pepper. As soon as it is thick pour over the fish, but do not let it boil; serve very hot.

OYSTER OMELETTE.—Make an omelette mixture with two yolks of fresh eggs, a tablespoonful of milk, a little pepper and salt, and the two whites of eggs whipped stiffly with a pinch of salt, melt one ounce of butter in a small omelette pan, pour the mixture in, and when it begins to set drop on the top five oysters that have been bearded, divided, and sprinkled with a little lemon juice. Cook the omelette for a few minutes, then put it just as it is in the pan in the oven to rise, which will take about five minutes. Turn out on a hot dish, fold it over, and serve as quickly as possible.

FRICASSEED OYSTERS.—These are very nourishing for those patients who are able to take cream. Make a quarter of a pint of single cream hot in a double boiler or in a galley-pot standing in a saucepan of boiling water. When the cream boils stir in a teaspoonful of flour that has been moistened with a little milk; season it with pepper and salt and a pinch of mace; stir till it boils, and let it cook for five minutes. Beard six or eight oysters and throw them into the sauce; allow them in just long enough to get plump (if left too long they get hard and shrivel up); have ready a slice of toast on a very hot dish, pour the oysters on this, and garnish with lemon sliced very thinly.

FILLETS OF SOLE.—Bat out the fillets of a perfectly fresh sole with a knife dipped in cold water; season them with pepper, salt, and sprinkle with lemon juice. Fold them over into kite shapes, and trim them neatly; place them in a a well-buttered tin, sprinkle again with lemon juice, and cover them with a buttered paper. Cook them in a moderate oven for twelve minutes. These should be prepared early, so that the bones of the sole can be made into stock for the sauce. Put them in a stewpan with a little salt, four peppercorns, and one onion sliced; cover with cold water, bring quickly to the boil, skim, and cook for half an hour, strain it, and put half a pint on to boil; then mix it on to half an ounce of cornflour that has been mixed with the pulp of a ripe tomato; stir altogether till it boils, adding two or three drops of carmine. When the fish is cooked, dish up and pour the strained sauce over it, and sprinkle with chopped parsley.

BOILED SOLE.—Select a medium-sized one, and lay it in cold salted water for half an hour, then put it in a very small fish kettle or stewpan with enough cold water to cover it, add the juice of half a lemon, and a pinch of salt; cover the fish with a piece of buttered paper, bring it gently to the boil, then simmer for a few minutes. Dish up on a hot dish, rub it over with a little fresh butter, sprinkle with chopped parsley, and pour the sauce round.

Fry together in a stewpan half an ounce each of butter and flour, when thoroughly mixed pour on to them a quarter of a pint of boiling water, add a little salt and one tablespoonful of lemon juice, and lastly one of cream, bring to a boil. Strain the sauce, pour it round the sole—not over it.

SOUCHET OF SOLE.—Put the sole in a stewpan and cover it with fish stock or water, add four peppercorns, a little salt, and one onion sliced, bring it to the boil, then draw to the side of the stove and simmer for eight or ten minutes; take up the fish and keep hot between two plates. Strain the liquor, and to one pint add the whites of two raw eggs, and whip them together, reboil the stock and simmer till it clears, strain it through a soup cloth, reheat it. Put the fish in a tureen or deep dish, pour the stock over it, sprinkle with chopped parsley, and serve as hot as possible.

### A NURSE'S WEDDING.

AN interesting wedding lately took place at Datchet Parish Church. The bride was Miss Ada Thompson, a nurse at Princess Christian's Private Nursing Home, Windsor, and the bridegroom Mr. Frank Hay Roberts. The Dean of Windsor, assisted by Canon Thompson, performed the ceremony, and the Princess Christian was present and signed the register. Her Royal Highness also gave the bride a Queen Anne silver tea set. Miss Thompson was trained at St. Bartholomew's Hospital, Rochester, after which she spent two years at the Royal National Hospital for Paralysis, Queen's Square, Bloomsbury, and then entered upon her work at the Princess Christian's Private Nurses' Home at Windsor, which she relinquishes on her marriage.

### Hampshire Unions in Conference at Winchester.

Nothing but good can come of such conferences as that held in the Board-room of Winchester New Union some little time ago. Mr. W. Shenton, of Winchester, took the chair, and the Local Government District Inspector, Mr. Baldwin Fleming, read a valuable and exhaustive paper on "Nursing in Workhouses." He began by dividing his subjects into two heads—what workhouse nursing was and what it was not, and cleared away any ground of misapprehension by defining the difference between trained and untrained nursing. He regarded training as essential, although he did not consider a nurse experienced in nursing was incompetent because untrained. Having thus explained himself, he gave a full account of the different unions in Hampshire, comparing number of patients and the proportion of nurses attendant on them, and followed this up by fully discussing the new Local Government Board order and the manner in which it affected all and each. The most serious and common defect in workhouses, Mr. Fleming considered, was the night nursing, for in a large number of workhouses the night nursing lies wholly in unskilled hands.

So much for the present state of affairs; now comes what it ought to be. In Mr. Fleming's opinion workhouses should be divided into three classes—first, those maintaining a regular staff; secondly, those requiring some skilled nursing, such as one skilled nurse with competent assistance is able to manage; and, thirdly, those small institutions which only need the occasional services of a skilled nurse. Every workhouse ought to have a spare room always ready for occasional use, and arrangements should be made with a nursing institution to supply a trained nurse whenever required. By these provident means a skilled nurse could be beside her patient in the course of a few hours.

The friction frequently occurring between matron and nurse arose, in the speaker's opinion, from a misunderstanding. Each in reality was the other's best help, and unfortunately, the Press in writing on these topics had accentuated the trouble by unwittingly misrepresenting the functions of each and their bearing one upon another. One difficulty was that the salary of the matron was in some instances too low. Amongst the principles Mr. Fleming insisted upon was the value and economy of efficient nursing, and the extravagance of making a skilled nurse a hewer of wood and scrubber of floors and a drawer of water. He pointed out the importance of fresh air and hot water and all the paraphernalia incident to the needs of sickness and the folly of allowing good work done during the day by a competent nurse to be undone at night by incompetent labour. He laid stress upon the proper supply of material comforts to keep the physical machinery of the nurse in perfect working order. Under this head was included diet, amount of work that could be done, and stated his objection to continuous night duty.

Mr. Fleming paid a tribute to the work of the Workhouse Infirmary Nursing Association, and also to the excellent care many of the untrained matrons had exercised on behalf of the infirm before the advent of the skilled nurse. In the discussion that followed he informed his audience that the Local Government Board had under consideration the question of waste and unsuitable food.

The whole paper showed the careful study that had been bestowed upon the subject, and nurses have to thank Mr. Fleming for the great and discriminating sympathy and insight he has displayed in regard to their interests.

### FOR THE CRIPPLED.

Alderman Treloar is organising a scheme to provide 5,000 poor crippled children living in London with hampers at Christmas. Anticipation runs high, no doubt, on the present occasion amongst likely recipients. H.R.H. the Prince of Wales has sent £5 to this fund.

### The Workhouse Infirmary Nursing Association.

A meeting of the Workhouse Infirmary Nursing Association was held in St. Martin's Town Hall, Mr. E. Boulnois, M.P., in the chair.

The proceedings opened by voting a message of condolence to H.R.H. the Duke of Teck upon his late bereavement, the Princess Mary, Duchess of Teck, having been President of the Association.

Miss Wilson (hon. secretary) made a statement of the work achieved and that undone. Then two resolutions were proposed, discussed, and finally put to the meeting.

The first, proposed by Mr. Bousfield, seconded by Lord Montagu of Beaulieu, and carried by eight votes to seven (only very few present voted), was: "That the Workhouse Infirmary Nursing Association is of opinion that the time has arrived when the work of the association must of necessity cease."

The second, carried unanimously, was: "That the Executive Committee and the office of the association shall continue for a time as a centre for information for guardians and nurses and for the maintenance of a connection with nurses and probationers now belonging to the association."

Towards the end of the meeting Miss Louisa Twining, one of the founders of the association (with Lady Lothian and Lady Montagu of Beaulieu; unavoidably absent from the meeting) gave an admirable digest of what had been accomplished during the last eighteen years in nursing the sick in workhouses. She pointed out that the association withdrew from its chosen work because with the Local Government Board order of last September, which prohibits pauper nursing in the workhouse, they had gained their cause, and therefore they now threw the responsibility of providing trained nurses for workhouses on to a wider and more central authority. Miss Twining declared that it was an anomaly for a private association to do the work of a State department; but if the Local Government Board would have accepted the overture made them by the association, if it would have given them a recognised position, and granted them some money, the association would have braced itself to meet the new calls made upon it. Miss Twining further remarked that defective as the order was, yet those who, like herself, had seen what things were in the past, could only feel deeply thankful that so much had been accomplished.

The discussion was full of sympathy with the committee, and appreciation of work done by the association. The burden of all the speeches by delegates of Guardians and those who were interested for the nurses, was "Please go on with your work." Lady Lothian, one of the founders, made an almost passionate appeal for the work to be continued. The numerous dangers that are still to be overcome, the probability that a retrograde movement would set in from the difficulty of obtaining trained nurses, the injustice of engaging young girls to nurse the scum of the population, and of, as in many country workhouses, giving them no opportunities of training and thus condemning them to inferiority for life, were all brought forward.

Lord Montagu of Beaulieu and Miss Twining, however, stated their own case too clearly not to justify their action. Three things are needed—money first, nurses and suitable candidates for training second, and some official recognition from the Local Government Board, third. The money might be obtained; nurses, however, could not unless the terms of service were made more attractive, and the Local Government Board was polite but indifferent towards the association. Under the circumstances the only thing was for the association to retire from the work.

The meeting closed with the usual votes of thanks.

### Nursing Directories.

The *Nursing Record* having stated that the issue of "Burdett's Official Nursing Directory" constitutes a "public danger," it becomes desirable to compare the various directories which at the present time exist, and to ascertain which of them best fulfils the requirements of nurses and the public, for the fact must not be hidden that if a directory is to serve its proper purpose it must be arranged to meet the wants of the medical profession and the public as well as those of the nurses themselves. Ostensibly there are three directories at present available, namely, "Burdett's Official Nursing Directory," the "Register of Trained Nurses" issued by the Royal British Nurses' Association, and the "Nursing Directory," a publication which is stated to be issued under the authority of the Matrons' Council, and is published by the *Nursing Record*. We have the authority of last week's *Nursing Record* for the statement that in January, 1896, the "Matrons' Council" decided to appoint a committee to edit the "Nursing Directory," but whether such a committee has ever been actually appointed, and of whom the Matrons' Council itself consists, there is nothing to show either in the Directory or in the paper referred to. Not that it matters much, because, in fact, we have only to deal with two directories, one the Register of the Royal British Nurses' Association, from which the "Nursing Directory" seems to be principally compiled, the other "Burdett's Official Nursing Directory."

To begin with the older publication, we first observe that in the preface it is stated that "at present any woman, although she may be destitute of knowledge, or of moral character, or of both, can without let or hindrance term herself a trained nurse, and can obtain employment in that capacity." Then the preface goes on to describe how the Register has been formed and the sort of people who are on it, and it further states that "women who had been for three years engaged in nursing the sick—whether trained in hospitals or not—were for the first six months held to be eligible for registration. At the end of that 'period of grace' three years' hospital service was made an essential condition;" so that unless the Register has since then been sorted out again and again, and its issues properly supervised, where is the security that it does not now contain the names of the very people who, as it says in the preface, "bring about much danger to the sick and much discredit to the calling"? Evidently the whole value of the Register as an annual directory depends on the regular and repeated investigation of the present status of the nurses.

In the last edition of the Register, it is confessed that it has been decided that even the "verification slip" which used to be sent annually to each nurse shall be discontinued; so that this Register is now issued in such a fashion that the editors do not take any means to find out even whether the persons entered on its pages are yet alive!

Now what about "Burdett's Official Nursing Directory," the issue of which, according to the *Nursing Record*, is a "public danger"? In it we have the assurance of Sir Henry Burdett that what it does is merely to state "the facts as nearly as they can be ascertained. Anyone possessing this Directory can ascertain the experience or training of each nurse whose name appears in it." What more do people want? It surely cannot be a "public danger" to speak the truth.

The "danger" is not to the public, but to the continued existence of the Directory which is issued by the *Nursing Record* under the title of the "Nursing Directory." When this directory speaks of the nurses whose names appear on its pages as having received "definite professional recognition," we may well ask, Who are the members of this Matrons' Council under whose "authority and supervision" they are enrolled, and to what great hospitals are these ladies attached, that they should arrogate to themselves the right to say what are "recognised" training schools, and who is a trained nurse and who is not? The thing is absurd.

The "profession of nursing" is a far wider thing than is dreamed of by those who imagine that only those who have been trained in "recognised" institutions have the right to call themselves "trained nurses"; and if a demand has arisen for a directory which shall be free from the taint of cliquism which attaches to the Matrons' Council, these ladies and those who pull the strings to which they dance have only themselves to blame.

The *Nursing Record* is most amusing when it enters on the field of criticism. Speaking of the Official Directory it says: "When it is recognised that large numbers of names appear in it, with qualifications attached, without the consent of the nurses themselves, and in consequence without any verification of the entry, the public danger of such a directory becomes at once apparent." This is particularly neat, because the very essence of Burdett's Official Nursing Directory seems to be that the information is not dependent on the statement of the nurses themselves, but is stated to be verified from official or independent sources. Herein lies its value, both to the public and the nurses, its statements being the result of an independent investigation of the qualifications claimed.

And now we come to the amusing thing. After careful search the editor of the *Nursing Record* has found two errors, or what she pretends to be errors, in "Burdett's Directory." Two ladies are described in the latter as engaged in private nursing, and as living at 12, Holles Street, whereas, according to the *Nursing Record*, they are both engaged at the Gordon House Home Hospital; and in regard to one of them we are told that she "has never been engaged in private nursing." But what is the Gordon House Home Hospital? Well, it is No. 12, Holles Street; and if it is not a private nursing home, and if the nurses there employed are not engaged in private nursing, we fail to understand the English language. Here comes in the true feminine editor. She searches the new directory through and through, and the only error which she specifies is one which is not an error at all. The interests of the nursing profession as a whole are as nothing when employment in her own little private nursing home is spoken of as private nursing! Then, indeed, the torrent of wrath is poured out. Whether Sir Henry Burdett will be submerged by it or not we cannot say. Perhaps he will rise from his trials with a chastened spirit, ready to admit that even No. 12, Holles Street, is a public institution! But we cannot but feel that both the public and the nurses will prefer a directory issued by an entirely independent authority to one tainted by the advocacy of a journal which so evidently has an axe to grind. The "public danger" does not arise from the issue of an Official Nursing Directory by an outside authority, but in the using of the form of a directory for the sake of giving power and influence to a clique.

### Novelties for Nurses.

### FOR THE NURSERY.

Any material which helps to lessen the tendency that the delicate skin of an infant has towards chafing is certain to secure the support and approval of the careful mother and nurse. Messrs. C. H. Wolstenholme and Co., 22, Tib Street, Manchester, are the patentees of a patent swansdown diaper, called the "Hygroscopic," which is perfectly absorbent, and delightfully soft. From a sample received we have no hesitation in pronouncing it to be one of the most useful inventions for the purpose we have seen, and can strongly recommend a trial of its virtues. A specimen will be sent on receipt of seven stamps, the price being somewhat under that of the ordinary linen diaper, although it has the advantage of being very durable.

### Everybody's Opinion.

[Correspondence on all subjects is invited, but we cannot in any way be responsible for the opinions expressed by our correspondents. No communication can be entertained if the name and address of the correspondent is not given, as a guarantee of good faith but not necessarily for publication, or unless one side of the paper only is written on.]

### HOUSEKEEPING.

"A CONSTANT READER" writes: I should be so glad to know from your correspondent "M. D." how she manages to live without meat or vegetables. No mention is made of either in her expenditure. "M. D." could not possibly live such a hard life on only "tea," coffee, cocoa, and Quaker oats.

### A DISTRICT NURSE'S PAY.

"M. R." writes: I am very fond of reading, especially "The Mirror" of your paper, but could not help feeling a bit cross when reading "M. D.'s" note on November 27th. I think those sort of letters tend to bring down our salaries. Committees, I dare say, read THE HOSPITAL as keenly as we do, and then they may say, "Here's a nurse for £60, why need we advertise £70." Then surely "M. D." does not live on bread and butter and Quaker oats. There is nothing down in her list for meat, bacon, vegetables, eggs, or light, and what about the "tenth." I find collections, subscriptions, and what I call "church expenses" a serious item. I am a district nurse and have £80 a year, and have not felt overdone yet with my money. I think we all ought to try and raise salaries and not tempt people to offer small ones. Surely the labourer is worthy of his hire.

*** We quite agree. A salary of less than £65, with lodgings, is too low.—ED. *T. H.*

### THE ROYAL BRITISH NURSES' ASSOCIATION.

DR. BEZLY THORNE writes: In view of the influence which the result of the approaching general meeting of the Royal British Nurses' Association must have on the future of that body, I venture to ask you to allow me to make a plain statement on a subject which has been very seriously misrepresented. It is the more important that I should do so as for some two years past misstatements on the subject have been sedulously and persistently diffused, and as the editorial mind of THE HOSPITAL has altogether escaped the poison, as shown by the leading article of a recent issue. I refer to the retirement in rotation of the matrons in batches of one-third from the General Council. It has been stated that that provision has been carried out as the result of a trick or a betrayal on the part of the honorary officers of the Corporation, and that they, in so acting, have inflicted an unmerited insult on the matrons of some of the more important hospitals of the metropolis and of the provinces. The facts are these: *Ex-officio* seats were apportioned to the matrons of certain training schools when the bye-laws and regulations of the then unincorporated Association were drawn up. Subsequently it was felt that the creation of permanent members of the governing body was a measure fraught with danger, and at a meeting of the Executive Committee held on October 13th, 1891, it was proposed that at least one-third of the matrons and one-third of the sisters and nurses of the General Council should retire annually by rotation, and not be eligible for re-election until the following year. On November 2nd of the same year Dr. Bedford Fenwick announced to the General Council that at the next meeting he would propose a resolution in that sense, and, in fact, on January 8th, 1892, he did propose, and Mr. Arthur Barker seconded, and it was carried, that a rule to the effect above stated should be added to the regulations of the Association. It was in full view of this fact, and in that sense, that the charter and bye-laws at present in existence were drawn up, submitted to, and approved by, the Lords of the Privy Council. But when the time came for the retirement in rotation of certain of the matrons, the execution of the provision in question was received with affected amazement. Appeal was made to Mr. Muir Mackenzie, the standing counsel of the Corporation, to Sir Richard Webster, and Mr. Swinfen Eady, Q.C., and all expressed unequivocally the opinion that compulsory triennial retirement in batches of one third was clearly ordained by the bye-laws as approved by the Privy Council. It is by means of such unblushing misrepresentations as I have indicated that the agitation fomented by a small section within the Corporation has been maintained for the last two or three years, and I hope that, in view of the approaching important meeting, the members will clear their minds of all misapprehension on the subject. I will only add that it is my earnest hope that the salutary provision of triennial retirement will be maintained intact in the new bye-laws which it is proposed to lay before the Privy Council for approval. With that object in view, I have already given notice that at the general meeting I shall propose amendments to the draft of the bye-laws relating to that subject. In all other respects the new scheme of bye-laws has my hearty approval.

"A CONSTANT READER" writes: I shall feel grateful if you can find room in your very well-read paper for the following remarks to my fellow-nurses who are members of the above. I wish to ask them all to carefully read the new bye-laws which have been sent to us, and also the very clear statement concerning them, for I take it that many of us knew little about bye-laws before. I did not, and therefore now that we have them made so clear to us we ought to study them, and if we approve of them to vote for them and get them passed. You will see that they cannot be passed unless the majority of our members vote for them. It seems to me they are a step in the right direction, for do they not sanction nurse-members on the General Council as well as matrons and doctors? No one can say then that we do not have a voice in our own affairs. Of course the nurse-members will be chosen from those whose experience in nursing makes them suitable, and I do not think the matrons will feel so humiliated as it was stated some time ago (if the working subordinates were on the Council) they would be. For is not one nurse of experience as good as another? And what are our matrons but trained nurses of experience? And such we can all be in time if we wish, so that bye-law is greatly to our advantage. Our Royal President has also sanctioned them and hopes that we shall give our vote and get them passed. I am glad to see that in future we shall have our journal monthly—the small sum of 2s. per annum no one can object to, and we shall have a paper of our own, which will have no quarrelling letters in. I think it behoves all the members who have not paid up their subscriptions to do so at once, for soon we shall be flourishing again and the subscriptions will go up, and it will be vexing to be out of the membership just as we are starting afresh, and have to pay larger fees and begin again. I want to say, too, that we shall never really realise what we owe to our hon. officers; but for their timely help and unceasing efforts we must have failed in much. They have given their valuable time and money for our affairs, and got us nearly, if not quite, over a most trying time for our association; and we cannot be too grateful to them for all they have done. I hope our general meeting will be well attended, that our bye-laws will be passed, and that with the New Year we shall start a really good and prosperous future for the R.B.N.A. I am only a simple member, and none of the officials know that I am writing this. I have watched the affairs of the association for two years and read both nursing papers, and I feel I should like to say this to my fellow nurses—"God speed to our R.B.N.A."

### A MENTAL ATTENDANT'S OPINIONS.

"A MENTAL NURSE" writes: It is a lamentable absurdity that young women who spend a goodly portion of their lives in hospitals for mental diseases, receiving training in the tactful management and the nursing of the insane, on coming out into the world, to make this intricate, if not the most trying and melancholy branch of the whole nursing profession, their calling, are not only depreciated, but they are refused the same recognition and privileges extended to their more valued sisters in the medical and surgical line. They cannot be allowed membership at the Royal British Nurses' Association, so far as one lady on that council is concerned, at any rate. Mrs. Bedford Fenwick has said that it would be a disgrace for the R.B.N.A. to admit as members women who devoted their lives to the cause of the insane, otherwise mental nurses. I feel sure that the good families of London and elsewhere who, unfortunately, have needed and tried their services, with the physicians in attendance, and who should be best judges, will not agree with this. Nevertheless, it will be a revelation to me if anyone, through your valuable paper, could point out wherein lies disgrace in making a speciality of nursing mental disorders any more than those of fevers or a broken limb. Why should it not be considered as honourable. It savours of the mediæval half savage state of civilisation, for then it was the poor insane patients, now it is the young women who elect to take charge of them that are held up to public contempt as something to be frowned down and avoided. I could not imagine anybody who, truly knowing the character of mental nurses' duties year in year out, and the mental strain consequently only too often involved, having the conscience to throw a straw in their way. If Mrs. Bedford Fenwick had spent a few years of her life as matron in one of our large asylums instead of at St. Bartholomew's Hospital it might not be unreasonable to suppose that she would be advocating the services of a competent mental nurse as being the best and most correct for surgical operations. Therefore I venture to hope, even if I have no influence, that any person or persons capable of drawing so narrow and odious a comparison shall be for ever removed from a council for promoting the welfare of nurses as being incapable of judging what is for the best interests of everyone concerned.

### Minor Appointments.

BELPER WORKHOUSE.—Miss Kate Nichols, late of St. Mary's Hospital, Paddington, was appointed on December 4th Superintendent Nurse of this institution. She was trained at Birmingham Workhouse, and subsequently held appointments in that infirmary and in the Hospital for Women, Brighton.

ROYAL HOSPITAL FOR SICK WOMEN AND CHILDREN.—Miss Golding, who was trained at the Liverpool Royal Infirmary, and who during the subsequent five years had great experience as temporary ward sister, theatre nurse, and night superintendent, has been appointed Sister of this hospital.

THE CONVALESCENT HOME FOR CHILDREN, CLIFTON ROAD, WESTON-SUPER-MARE.—Miss Clark, trained at Edinburgh Royal Infirmary, and for six years sister to the children's medical floor in the Royal Hospital for Sick Women and Children, Bristol, has been appointed Matron of this institution.

ST. OLAVE'S INFIRMARY, ROTHERHITHE.—The new Charge Nurses are Miss Louisa Whittington, who was trained and subsequently nurse and charge nurse at Middlesex Hospital, and Miss Kate Fowler, who was trained and afterwards nurse at the Wandsworth and Clapham Union Infirmary.

OTTERSHAW WORKHOUSE INFIRMARY.—Miss Ellen Ringrose is the new Superintendent. She was a probationer at South Devon and East Cornwall Infirmary, and subsequently charge nurse, St. George's-in-the-East Infirmary.

INFECTIOUS DISEASE HOSPITAL, BOOTLE.—After four years' private work, Miss Honorah Foley takes up work in the above hospital as Charge Nurse. She was trained at Widnes Fever Hospital.

BROMPTON CONSUMPTION HOSPITAL.—Nurse Edith Joseph, trained at the Royal United Hospital, Bath, has been appointed staff nurse at this hospital.

### Wants and Workers.

[THE attention of correspondents is directed to the fact that "Helps in Sickness and to Health" (Scientific Press, 28 & 29, Southampton Street, Strand, London, W.C.) will enable them promptly to find the most suitable accommodation for difficult or special cases.]

THE Matron of a small isolation hospital would be thankful to receive a piano to help her in holding a service with the patients on Sundays. She would pay carriage and a small sum. Address Matron E. V. L., to the care of the Editor THE HOSPITAL.

### Notes and Queries.

**The contents of the Editor's Letter-box have now reached such unwieldy proportions that it has become necessary to establish a hard and fast rule regarding Answers to Correspondents. In future, all questions requiring replies will continue to be answered in this column without any fee. If an answer is required by letter, a fee of half-a-crown must be enclosed with the note containing the enquiry. We are always pleased to help our numerous correspondents to the fullest extent, and we can trust them to sympathise in the overwhelming amount of writing which makes the new rules a necessity. Every communication must be accompanied by the writer's name and address, otherwise it will receive no attention.**

*Volunteer War Nursing.*

(86) Will you kindly oblige me by telling me if you know to whom I must apply to enrol my name on the list of nurses offering their services to nurse the sick and wounded in the present serious war in India?—*In Earnest.*

Write to the India Office, St. James's Park, S.W., for forms of application.

*Climate of Dominica.*

(87) I should be greatly obliged if you could inform me if the climate of Dominica is favourable to Europeans, and particularly for a consumptive patient.—*Inquirer.*

Dominica is not a particularly favourable climate for Europeans, and a consumptive patient must consult his medical attendant before going to live there.

*Medical Missionary.*

(88) Will you kindly tell me through THE HOSPITAL what is required of a trained nurse to qualify her for a medical missionary? Also where to apply for engagement as such.—*Nurse Lena.*

The Church of England Zenana Missionary Society, 9, Salisbury Square Fleet Street.

*Surgical and Nursing Homes.*

(89) Will you kindly send me a list of some of the best surgical homes and nursing institutions in London; or give me the name of a book where I could find them?—*Nurse Reid.*

See "Burdett's Official Nursing Directory," recently published, price 5s. 6d., post free.

*Considered Trained.*

(90) Will you kindly inform me if a nurse who has been trained at a well managed general hospital (containing 60 beds, daily average occupied 54) where a three years' certificate is given? Will she be considered a trained nurse, and will she be considered eligible for the same posts as nurses trained at larger hospitals?—*X.*

She is certainly trained, but if she wants to obtain one of the best posts she must extend her experience by becoming nurse at larger hospitals in first one class of work and then another.

*India and Stains.*

(91) Will you kindly tell me in which number of THE HOSPITAL there was a complete outfit for India given, and would you also let me know how to take stains out of ward mattresses, such as perspiration, &c.?—*S. J. Smyth.*

"The Mirror," October 3rd, 1896. You might try brushing them with Sapolio very slightly wet, and then rubbing them with a cloth wrung out of clean water, but probably the only thing will be to send the mattress to a cleaner's. Avoid such stains in future by putting a thick blanket between the sheet and mattress.

*The Fee for Massage.*

(92) I should be much obliged if you could inform me what the usual fee is for massage.—*A. S.*

From 5s. an hour. It varies greatly.

*Baby Farming.*

(93) Would you kindly tell me how I could get nurse children to begin baby farming? I am a hospital-trained nurse for many years, and through death I have been obliged to give it up.—*Mary G. Jane.*

Is baby farming a happy name? We suppose that you mean that you wish to have the care of infants, and, being a trained nurse, are especially qualified to look after them. But it is a risky business. We send you by post a copy of the Bill relating to such homes. After registering your house you must advertise and give your references to applicants, but you must take care that whatever you do is done openly under the eyes of responsible witnesses.

*Painful Feet.*

(94) I should feel grateful if you could tell me what to do for painful feet. I had to take a rest from hospital work, as I was growing flat-footed, and after six weeks' rest, and wearing surgical soles, they are still painful. I have been rubbing them with Elliman's Embrocation. Should I persevere with it or not? Ought I to bandage them or not?—*Agnes Lakeman.*

Feet may be painful from many causes. In some cases, from original malformation, a greater than normal strain is placed on certain ligaments, which in consequence become painful. Sometimes the occurrence of corns or chilblains leads people to stand in such attitudes that undue strain is thrown upon certain parts of the foot—pain, and even deformity, being thus produced. Far more frequently pain is due to the gradual development of flat foot, which, in turn, is mostly the result of rheumatic conditions; and, lastly, there is metatarsal neuralgia, or Morton's disease. It is clear then that "painful feet" require very careful consideration, and that our correspondent must be advised to consult a doctor who has had some experience in orthopædic surgery.

